# Nb Doping and Alloying of 2D WS_2_ by Atomic Layer Deposition for 2D Transition Metal Dichalcogenide Transistors and HER Electrocatalysts

**DOI:** 10.1021/acsanm.4c00094

**Published:** 2024-04-01

**Authors:** Jeff J. P. M. Schulpen, Cindy H. X. Lam, Rebecca A. Dawley, Ruixue Li, Lun Jin, Tao Ma, Wilhelmus M. M. Kessels, Steven J. Koester, Ageeth A. Bol

**Affiliations:** † Department of Applied Physics, Eindhoven University of Technology, P.O. Box 513, Eindhoven 5600 MB, The Netherlands; ‡ Department of Chemistry, University of Michigan, 930 N. University Avenue, Ann Arbor, Michigan 48109, United States; § Michigan Center for Materials Characterization, University of Michigan, 2800 Plymouth Road, Ann Arbor, Michigan 48109, United States; ∥ Department of Electrical and Computer Engineering, University of Minnesota, 200 Union Street Se, Minneapolis, Minnesota 55455, United States

**Keywords:** 2D materials, atomic layer deposition, WS_2_, doping, alloying

## Abstract

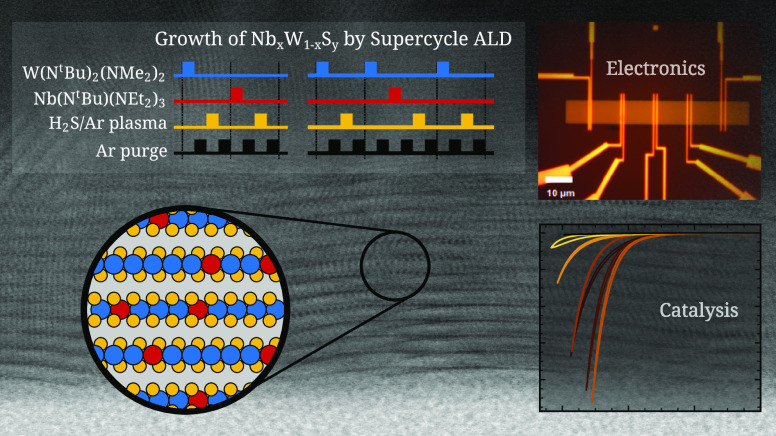

We utilize plasma-enhanced atomic layer deposition to synthesize two-dimensional Nb-doped WS_2_ and Nb_*x*_W_1–*x*_S_*y*_ alloys to expand the range of properties and improve the performance of 2D transition metal dichalcogenides for electronics and catalysis. Using a supercycle deposition process, films are prepared with compositions spanning the range from WS_2_ to NbS_3_. While the W-rich films form crystalline disulfides, the Nb-rich films form amorphous trisulfides. Through tuning the composition of the films, the electrical resistivity is reduced by 4 orders of magnitude compared to pure ALD-grown WS_2_. To produce Nb-doped WS_2_ films, we developed a separate ABC-type supercycle process in which a W precursor pulse precedes the Nb precursor pulse, thereby reducing the minimum Nb content of the film by a factor of 3 while maintaining a uniform distribution of the Nb dopant. Initial results are presented on the electrical and electrocatalytic performances of the films. Promisingly, the Nb_*x*_W_1–*x*_S_*y*_ films of 10 nm thickness and composition *x* ≈ 0.08 are p-type semiconductors and have a low contact resistivity of (8 ± 1) × 10^2^ Ω cm to Pd/Au contacts, demonstrating their potential use in contact engineering of 2D TMD transistors.

## Introduction

Two-dimensional transition metal dichalcogenides (2D TMDs) are a class of layered materials that have received widespread attention in both fundamental and applied research in recent years. The wide variety of properties of 2D TMDs makes them interesting for a broad range of applications, including electronics and electrocatalysis. To further engineer the properties of TMDs, doping or alloying is a valuable approach to obtaining desirable properties from different TMDs to be combined in a ternary material.

The development of 2D TMD-based transistors can benefit from the use of ternary TMD materials. Two of the major current challenges in achieving high-performance transistors based on 2D TMDs are achieving controllable doping and low contact resistivity. It has been shown that Nb doping can significantly improve the contact resistivity of WS_2_ monolayers^1^ and their performance as field-effect transistor (FET) channels.^2,3^

Electrocatalytic production of hydrogen is another important application that could benefit from the use of ternary TMD materials. This process currently relies on precious platinum catalysts for the hydrogen evolution reaction (HER) to proceed. While bulk MoS_2_ and WS_2_ are poor HER catalysts due to their inert basal plane,^4^ their edge sites are catalytically active.^5,6^ Hence, their catalytic activity can be increased by activating their basal plane and/or enhancing the density of edge sites. The engineering of WS_2_ edge sites using plasma-enhanced atomic layer deposition (PEALD) has recently been demonstrated.^7^ To further improve the catalytic activity of WS_2_, alloying or doping with Nb may be used to activate the basal plane.^8^ Nb and Ta are especially suitable dopants, as TMDs based on these metals have catalytically active basal planes.^9^

Synthesis of ternary Nb_*x*_W_1–*x*_S_2_ has previously been reported using various synthesis methods, as summarized in Table 1. Substitutional incorporation of Nb in WS_2_ has been demonstrated even up to high Nb/(Nb + W) concentrations of 40–60%.^10^ However, most studies focus on a lower Nb/(Nb + W) concentration of 0.1–20%. At these concentrations, incorporation of Nb in WS_2_ has been shown to yield tunable p-type conductivity,^1,3,11,12^ lowered Schottky barrier in FETs compared to pure WS_2_,^1,2^ tunable photoluminescence emission,^2,11,13,14^ and enhanced HER activity,^12^ although decreasing HER activity was also observed.^15^ In addition to the composition, the morphology of the deposited material is also important for applications. Both FETs and HER catalysis require the deposition of a film of material on (structured) substrates. Chemical vapor transport (CVT) produces bulk Nb_*x*_W_1–*x*_S_2_ crystals,^10,16−18^ such that further processing including exfoliation and transfer is required for application in FETs or catalysis. On the other hand, direct deposition of monolayer flakes and continuous thin films of Nb_*x*_W_1–*x*_S_2_ has been demonstrated by sulfurization,^3^ chemical vapor deposition (CVD),^1,2,8,11−14,19^ pulsed laser deposition (PLD),^20^ and ALD.^21^ Out of these techniques, CVD produces the highest-quality crystalline Nb_*x*_W_1–*x*_S_2_ monolayers, although their composition is sometimes observed to be nonhomogeneous.^3,14^ This can typically be attributed to differences in the volatility or reactivity of the Nb and W precursors used in the CVD process. Uniform growth of Nb_*x*_W_1–*x*_S_2_ on large-area substrates by ALD was reported by Yang et al.,^21^ although they could not determine the Nb content of the deposited films.

**Table 1:** Overview of the Literature on the Synthesis of Nb_*x*_W_1–*x*_S_2_^a^

synthesis method	morphology	Nb/(Nb + W)	notes	reference
CVT	platelets	0.5–5.0%	Nb effective as p-type dopant	Baglio 1983^16^
CVT	crystals	40–60%	phase separation at 500 K, alloy growth at 1300 K	Hemmat 2020^10^
CVT	sheets	10%	Nb ordered in atomic lines	Xia 2020^17^, Loh 2021^18^
heating of elemental mixture	powder	3.2%	unexpected negative impact Nb doping on HER catalytic performance	Chua 2016^15^
sulfurization (WO_*x*_ nanorods + Nb_2_O_5_ suspension)	nanotubes	10–20%	segregation of NbS_2_ phase when Nb > 20%	Zhu 2001^25^
sulfurization (drop-casted Na_2_WO_4_ + Nb(HC_2_O_4_)_5_)	monolayer islands	4.7–10.3%	inhomogeneous doping, demonstration *p*-FETs	Qin 2019^3^
CVD (NbO_2_, WO_2_, H_2_S)	monolayer islands	3–15%	heavy doping (15% Nb) decreases Schottky barrier, improves *p*-FET performance	Feng 2016^1^
CVD (NbCl_5_, WO_3_, S)	monolayer islands	6.7%	tunable bandgap (PL emission) by Nb doping	Gao 2016^13^
salt-assisted CVD (Nb, WO_3_, NaCl, S)	monolayer islands	0.5%	inhomogeneous doping, tunable PL	Sasaki 2016^14^
salt-assisted CVD (NbCl_5_, WO_3_, NaCl, S)	monolayer islands	0.5–1.4%	PL emission tuning saturates at 1.4% Nb, low Schottky barrier in *p*-FETs	Jin 2019^2^
salt-assisted CVD (NbO_*x*_, WO_3_, NaCl, S)	monolayer islands	7.3%	low contact resistance *p*-FETs, improved HER catalysis vs WS_2_	Pam 2019^8^
salt-assisted CVD (Nb_2_O_5_, WO_3_, NaCl, S)	monolayer islands	0.4–6.5%	doping-induced strain, good FETs (ambipolar or p-type) for Nb < ∼6.5%, tunable PL	Zhang 2020^11^
salt-assisted CVD (W,Cr,Fe,Nb,Mo)O_*x*_, (NaCl, S)	monolayer islands	0.10–0.17%	combined effects of various dopants, application to nonvolatile memory	Siao 2021^19^
salt-assisted CVD (NbCl_5_, WO_3_, NaCl, S)	monolayer islands	0.3–4.7%	improved HER catalysis vs WS_2_, ambipolar to p-type FETs with Nb concentration	Tang 2021^12^
PLD (Nb, WS_2_, S)	thin film	0.5–1.1%	decreasing conductivity from 0.5 to 1.1% Nb due to lower μ	Rathod 2018^20^
ALD (NbCl_5_, WCl_6_, HMDST)	thin film	unknown	“low” doping better for FET mobility	Yang 2021^21^
plasma ALD (TBTDEN, BTBMW, H_2_S plasma)	thin film	∼10–90%		this work

a CVT = chemical vapor transport, CVD = chemical vapor deposition, PLD = pulsed laser deposition, ALD = atomic layer deposition, HMDST = bis(trimethylsilyl)sulfide, TBTDEN = (*tert*-butylimido)tris(diethylamido)niobium, BTBMW = bis(tertbutylimino)-bis(dimethylamino)tungsten, FET = field-effect transistor, and HER = hydrogen evolution reaction.

ALD is particularly suited for the synthesis of compositionally uniform films due to its reliance on self-limiting surface reactions, which eliminate the flux dependence of film growth. Additionally, ALD can be used to accurately tune the composition and doping level of materials through the use of supercycle processes.^22^ ALD also allows for direct film deposition onto device substrates at low processing temperatures compared with common CVD synthesis approaches. Furthermore, we have recently shown that by tailoring the design of supercycle ALD processes, control over the atomic ordering of the ternary material can be obtained.^23^ This provides a novel avenue to tune the structure and properties of ternary materials.

In this work, we prepare thin films of ternary 2D TMD Nb_*x*_W_1–*x*_S_*y*_ films by plasma ALD. Widely tunable composition *x* is achieved through a supercycle process combining previously established ALD processes for WS_2_^7^ and NbS_3_.^24^ We discuss the chemical composition and structure of the films as a function of the NbS_3_/WS_2_ ALD cycle ratio. In order to achieve a lower Nb content for the growth of Nb-doped WS_2_, an additional ABC-type supercycle process is utilized. The electrical properties of the Nb-doped WS_2_ films are evaluated for application in field-effect transistors, and the performance of the alloyed films in hydrogen evolution catalysis is evaluated.

## Results and Discussion

**Figure 1:**
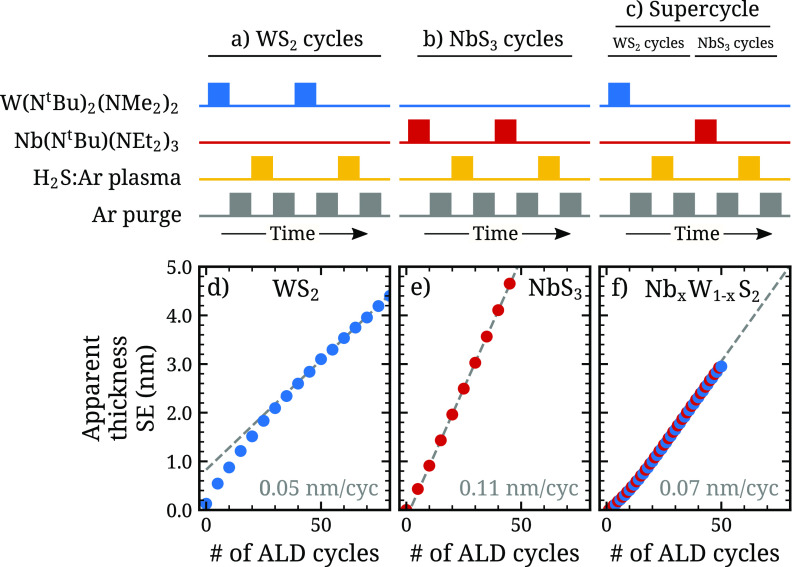
Growth of pure WS_2_ and NbS_3_ and ternary Nb_*x*_W_1–*x*_S_*y*_ by plasma ALD. The deposition processes are illustrated schematically in (a–c), and the evolution of the apparent film thicknesses measured by in situ spectroscopic ellipsometry is shown in (d–f). For the Nb_*x*_W_1–*x*_S_*y*_ growth shown in (f), a supercycle consisting of the 1 NbS_3_ cycle and the 1 WS_2_ cycle, i.e., 1Nb/1W, was used, as illustrated in (c). After the nucleation phase, all processes exhibit the linear growth typical for ALD. The growth per cycle (GPC) of NbS_3_ is higher than that of WS_2_, and the GPC of the alloy is intermediate with those of the two pure materials.

Figure 1 presents schematics of the ALD processes for the growth of WS_2_, NbS_3_, and ternary Nb_*x*_W_1–*x*_S_*y*_, as well as in-situ spectroscopic ellipsometry (SE) measurements of the growth of these materials onto SiO_2_. The ternary film was grown by alternating between ALD cycles of WS_2_ and NbS_3_, which is known as the supercycle method.^22^ The apparent thickness versus number of ALD cycles of both the pure and ternary TMDs exhibits the typical linear behavior after the nucleation phase, as expected from ALD. The self-limiting behavior of the pure WS_2_ ALD process has been previously reported.^7^ Also, the self-limiting behavior of a similar thermal (nonplasma) NbS_*x*_ ALD process at a reduced growth temperature of 250 °C has been previously reported.^26^ To validate the use of a higher growth temperature of 300 °C used in this work, it was verified through in situ spectroscopic ellipsometry measurements that no thermal decomposition of the Nb precursor occurs on the SiO_2_ substrate at this temperature. The growth per cycle (GPC) of NbS_3_ is significantly higher than that of WS_2_. Such a difference in GPC was also observed in previous reports.^7,26^ While the exact reason for this difference remains undetermined, it may be related to different bonding configurations of the W and Nb precursors on the surface. The growth per (super)cycle of the ternary film is intermediate with that of the two pure materials.

### XPS Analysis

**Figure 2:**
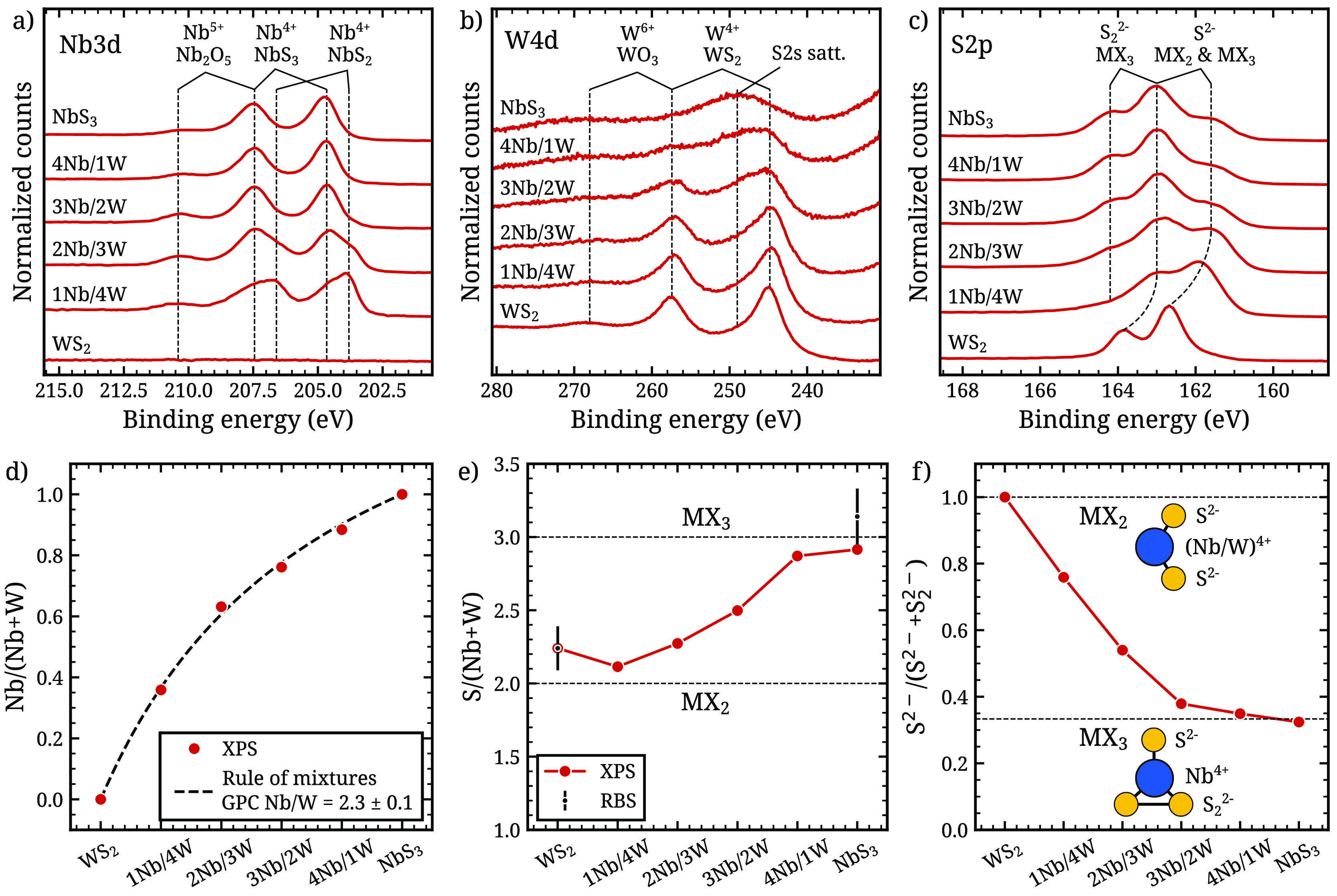
XPS elemental scans for orbitals Nb 3d (a), W 4d (b), and S 2p (c) with peak attributions which are discussed further in the main text, and derived composition parameters Nb/(Nb + W) (d), S/(Nb + W) (e), and S^2–^/(S^2–^+S_2_^2–^) (f) of the deposited films. In panels d and e, the relative W content obtained from XPS was scaled by a factor of 1.30 to agree with RBS measurements as explained in the main text. The insets in f show the bonding configuration in NbS_2_ and NbS_3_. Peak fitting results for all spectra are included in supplementary Figure S1 and Table S1.

The supercycle approach to growing ternary materials by ALD offers a straightforward method of tuning the composition of the film by changing the relative number of WS_2_ and NbS_3_ ALD cycles within the supercycle. To verify control over the composition in this way, a series of 6 samples with varying compositions was prepared. The samples were prepared using a supercycle process of *n* WS_2_ cycles followed by (5 – *n*) NbS_3_ cycles for *n* = 0, 1, 2, 3, 4, and 5. From high to low *n*, these samples are referred to as WS_2_, 1Nb/4W, 2Nb/3W, 3Nb/2W, 4Nb/1W, and NbS_3_. The total number of 5 ALD cycles per supercycle (i.e., the supercycle length) was chosen to ensure the amount of material deposited per supercycle does not exceed a monolayer, ensuring good mixing of Nb and W within the 2D layers.^23^ The number of supercycles was chosen such that the thickness of each film is approximately 10 nm, which was monitored using in situ spectroscopic ellipsometry. The composition analysis of the films was performed by X-ray photoelectron spectroscopy (XPS). Figure 2 shows the high-resolution scans of the Nb 3d, W 4d, and S 2p orbital regions. Peak fitting results of the XPS orbital scans discussed below are reported in supplementary Figure S1 and Table S1.

### Nb 3d Spectra

Figure 2a shows the Nb 3d scans. For the WS_2_ sample, no signal is observed in this spectral range, indicating no Nb contamination and the absence of any overlapping peaks from elements other than Nb in this spectral region. The Nb 3d spectrum of the NbS_3_ sample can be deconvoluted into two doublets (see supplementary Figure S1). The doublet at lower binding energy (BE) has the highest intensity and is attributed to niobium sulfide (Nb^4+^). In the literature, both NbS_2_ and NbS_3_ phases have been observed experimentally, both with Nb in a 4+ oxidation state.^24^ Which of these phases is present in these samples is discussed in more detail below. The other minor doublet at higher BE can be attributed to Nb^5+^, indicating partial oxidation of the film (i.e., the formation of Nb_2_O_5_).^27^ This assignment is also supported by XPS measurements taken on the same sample a month later (not shown), which revealed that the doublet at higher BE increased in relative intensity due to further oxidation over time. The Nb 3d spectra of the other Nb-rich films 4Nb/1W and 3Nb/2W are qualitatively similar to the spectrum of the pure NbS_3_ sample. On the other hand, the W-rich films 2Nb/3W and 1Nb/4W exhibit an additional doublet in the Nb 3d spectrum at a lower BE relative to the Nb^4+^ doublet. This indicates the presence of another bonding environment for Nb in these films. As will be shown below in the discussion of the S 2p spectra, this new Nb 3d doublet originates from Nb^4+^ in a NbS_2_ environment, whereas the other doublet originates from Nb^4+^ in a NbS_3_ environment.

### W 4d Spectra

Although the W 4f orbital is the most prominent W orbital in XPS, its peaks overlap with those of the Nb 4p orbital. Hence, we instead used the W 4d spectrum for the quantification of the W content. This spectrum also suffers from an overlapping peak, i.e., a satellite peak from the S 2s orbital, as can be seen in the scan of the NbS_3_ sample in Figure 2b. This peak was included in the spectral deconvolution, and its area was accounted for in the quantification of the W content. In contrast to the Nb 3d spectrum, no qualitative differences in the W 4d spectrum are observed as a function of the composition of the films. The main doublet in this spectrum is assigned to W^4+^ (WS_2_). A minor doublet can be observed toward higher binding energy, which is assigned to W^6+^ (WO_3_).

### S 2p Spectra

Scans of the S 2p orbital are shown in Figure 2c. For the WS_2_ film, one S 2p doublet is observed as expected for a pure WS_2_ phase. In the spectrum of the NbS_3_ sample, two doublets are observed, indicating the presence of two different bonding environments of sulfur in this film. We attribute these to S^2–^ (present in both NbS_2_ and NbS_3_) and S_2_^2–^ (present only in NbS_3_). For the pure NbS_3_ film, the fraction of sulfur in a S^2–^ bonding configuration approaches 1/3, which is the expected value for pure NbS_3_ (see Figure 2f). Hence, we observe a transition from a pure disulfide WS_2_ film to a pure trisulfide NbS_3_ film. For the ternary films, the S^2–^/S_2_^2–^ ratio indicates a mixture of disulfide and trisulfide phases.

### W Quantification

Using the Thermo Scientific modified Scofield relative sensitivity factors (RSFs), an S/W ratio of ∼3 is obtained for the WS_2_ sample. However, the single doublet in the S 2p spectrum of this sample, as well as the characteristic Raman signal (see below), suggests that it is pure WS_2_. This discrepancy suggests the underestimation of the W content in the XPS analysis. Upon comparison of W quantification using the W 4f orbital and the W 4d orbital for the pure WS_2_ sample, where both can be used without overlapping peaks, a ∼20% higher W content was found when using the W 4f orbital for quantification instead of the W 4d orbital, confirming the underestimation of W content based on the W 4d orbital. To verify the true W content of the samples, independent composition measurements using Rutherford backscattering spectrometry (RBS) were performed on WS_2_ and NbS_3_ samples. The RBS spectra are included in the Supporting Information. RBS compositional analysis provides an avenue for obtaining highly accurate stoichiometric ratios without the use of sensitivity factors or calibration standards, which are needed for XPS and can vary from source to source.^28−31^ In addition, quantitative accuracy limitations of XPS can arise from the inherent nature of the technique, where many factors, including shake-up effects, instrument artifacts, and multiplet splitting, can contribute to peak areas used for quantification and even vary among different atomic orbitals (i.e., W 4d vs W 4f).^32,33^ Oftentimes, RBS is used to complement XPS results,^34,35^ and in some instances, it is used as a benchmark for stoichiometric ratio determination,^36^ and sensitivity factors,^37,38^ such as RBS, is quantitative without the need for calibration standards.^28−31^ In contrast to XPS analysis, RBS measurements yield a S/W ratio for the pure WS_2_ sample of 2.24 ± 0.15. This suggests that the film is slightly sulfur-rich WS_2_. The S/Nb ratio of the NbS_3_ sample from RBS is 3.14 ± 0.19, which is consistent with XPS. This confirms that the W content is underestimated by XPS analysis of the W 4d orbital. In the rest of this work, the W content of films as derived from XPS of W 4d spectra was multiplied by 1.30 to agree with the RBS results.

### Nb/W Ratio

The compositions of *x* = Nb/(Nb + W) of the deposited films are shown in Figure 2d. It is found that the composition trend can be accurately described by the rule of mixtures,^22^ indicating that there is negligible interaction between the NbS_3_ and WS_2_ cycles in the process, resulting in highly predictable and reproducible film composition determined by the supercycle design.

### Film Purity and Oxidation

Negligible amounts of nitrogen were present in the films based on the N 1s scans (Supporting Information), indicating the complete removal of the ligands from the metal–organic precursors used for the ALD process. Significant counts in the C 1s region (Supporting Information) are observed and attributed to adventitious carbon. This attribution is supported by our previous work on an identical WS_2_ PEALD process, where the disappearance of the C 1s XPS signal following 40 s of Ar^+^ sputtering was demonstrated.^7^ In the Nb 3d and W 4d spectra, the presence of some oxide in the films can be identified by the presence of a doublet at a higher binding energy than that attributed to the sulfide. Approximately 7 thin film unit (TFU) (12–15 at. %) hydrogen was detected in all films by elastic recoil detection (ERD) analysis. The impacts of hydrogen in the films are discussed in more detail in the discussion of electrical characterization below.

### Advanced Supercycles for Accessing the Low-Nb Regime

**Figure 3:**
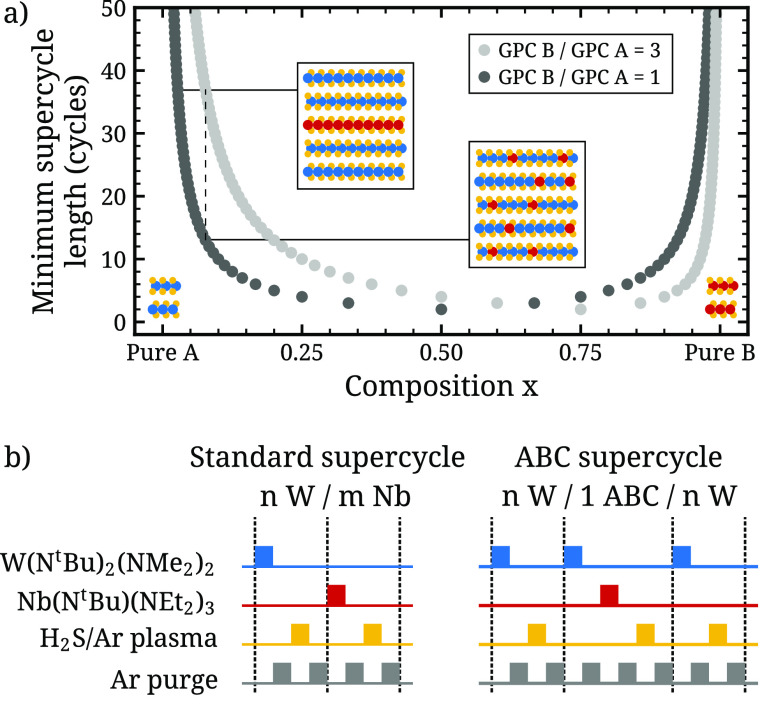
Schematic illustration (a) of the limited nanoscale homogeneity of an alloy film that may be obtained when there is a significant difference in GPC between the constituent materials A and B. If the GPC of B is high compared to that of A (dark markers), obtaining an alloy with a low amount of B requires following each cycle of B with many cycles of A. Such long supercycles lead to nanolaminate formation, as illustrated. On the other hand, when the GPC of A and B are similar (light markers), it is possible to obtain more homogeneous mixing of the alloy at low-A or low-B compositions as indicated with the dashed line. (b) ABC supercycle approach to reduce the GPC of NbS_3_ by replacing the NbS_3_ cycle with an “ABC” cycle where the Nb precursor dose is preceded by a W precursor dose, which adsorbs onto the sample surface and limits the available surface sites for Nb precursor adsorption.

It can be seen from the XPS data in Figure 2d that the alloy film with the lowest Nb content in this sample series, i.e., 1Nb/4W, already leads to a high Nb content of Nb/(Nb + W) > 35%. This can be attributed to the larger amount of material deposited per NbS_3_ cycle compared to the WS_2_ cycles (see Figure 1). In order to synthesize films with lower Nb content, longer supercycles could be used to further dilute the Nb. For example, a 1Nb/30W process would lead to a Nb/(Nb + W) fraction of 0.10 based on the rule of mixtures fit in Figure 2d. However, the amount of material deposited during such an extended supercycle will exceed a monolayer (∼0.6 nm thickness) such that the deposited film will no longer be a well-mixed alloy, but rather become a heterogeneous, nanolaminate film.^22^ This problem is caused by the difference in GPC between the NbS_3_ process and the WS_2_ process, as illustrated in Figure 3a. The larger the ratio of the GPCs, the longer supercycles are needed to obtain a low Nb content. Hence, a method of reducing the GPC of the NbS_3_ process is desirable, such that shorter supercycles can be used to obtain films with low Nb content while maintaining a homogeneous distribution of Nb.

We now modify the deposition process in order to reduce the amount of Nb incorporated per NbS_3_ cycle. Instead of the previously discussed NbS_3_ ALD cycles, we now use the so-called ABC cycles (Figure 3b), which consist of a W precursor dose, purge, Nb precursor dose, purge, H_2_S plasma, and purge. The durations of all steps are unchanged with respect to the previously discussed WS_2_ and NbS_3_ cycles. After the W precursor pulse, the substrate surface is saturated with adsorbed W precursor. This should limit the density of available adsorption sites for the Nb precursor, lowering the amount of Nb deposited per cycle.

To test the effectiveness of this method, a Nb_*x*_W_1–*x*_S_*y*_ sample was prepared using a 4*W*/1ABC/4W supercycle (see Figure 3b). For comparison, a second sample was prepared using the analogous standard supercycle process 4W/1Nb/4W (i.e., 1Nb/8W but shifted such that the first and last cycles are WS_2_). XPS analysis of the Nb 3d and W 4d regions of both films yields a Nb/(Nb + W) fraction of 0.25 for the standard supercycle process and 0.12 for the ABC supercycle process. Since the ABC cycle also incorporates W, this reduction in Nb content is not purely due to less Nb but also due to more W in the film. Since both samples were measured in the same batch, any difference in Nb 3d counts between the samples suggests a difference in Nb content since differences in, e.g., X-ray intensity are expected to be small. The Nb 3d peak area of the ABC supercycle sample is only 55% of its value for the standard supercycle sample. This indicates that the adsorption of TBTDEN during the ABC cycles is reduced by approximately 45% compared to that during the standard NbS_3_ cycles.

Having established the effectiveness of the ABC cycles for reducing Nb incorporation in the films, we now investigate whether the adsorption of TBTDEN in the ABC cycle still exhibits self-limiting behavior. Another 3 samples were prepared using the 4W/1ABC/4W process with Nb dose times in the ABC cycle of 10 (standard), 15, and 20 s. Table 2 compares the Nb content and XPS areas of both W and Nb signals for a standard 4W/1Nb supercycle with ABC supercycle type samples of varying Nb dose times. It is observed that the area of the Nb 3d peaks is largest for the largest Nb precursor dose time, while the W 4d area of this sample is the lowest. This suggests that less W is incorporated into the film when the Nb precursor dose time in the ABC cycles is extended. This can likely be attributed to the displacement of adsorbed W precursor molecules during the Nb precursor dose in the ABC cycle. The Nb/(Nb + W) fraction is 0.29 for the sample prepared with 20 s Nb precursor doses, which is comparable to the composition of the standard supercycle process, which is 0.25, as discussed above. This suggests that the displacement of the adsorbed W precursor by the Nb precursor may have been fully completed after an extended Nb precursor dose time of 20 s. However, saturation of the Nb content with respect to the Nb dose time is not yet observed after 20 s of dose time, and experiments with a further extended dose time would be needed to ascertain the saturation behavior of the ABC cycles.

**Table 2:** Effect of Nb Precursor Dose Time in the ABC Supercycles (See Figure 3b) on the Nb and W Content in the Resulting Films

	Nb 3d5 peak area (counts eV/ms)	W 4d5 peak area (counts eV/ms)	C 1s peak area (counts eV/ms)	relative Nb 3d5 area	relative W 4d area	Nb/(Nb + W)
4W/1ABC/4W @ 10 s Nb precursor	17.0	155	19.0	1	1	0.17
4W/1ABC/4W @ 15 s Nb precursor	22.7	150	17.5	1.33	0.97	0.22
4W/1ABC/4W @ 20 s Nb precursor	28.9	137	19.8	1.70	0.88	0.29
4W/1Nb/4W (no ABC)				-	-	0.25

In summary, replacing the NbS_3_ cycles in the supercycle process with ABC cycles, where the Nb precursor pulse is preceded by a W precursor pulse, effectively reduces the GPC of the NbS_3_ cycles by half. This allows Nb/(Nb + W) fractions to drop to ∼0.1 while still maintaining intralayer mixing of Nb and W,^23^ which is a factor of 2 lower than can be obtained using the standard supercycle recipe.

### Electrical Resistivity

**Figure 4:**
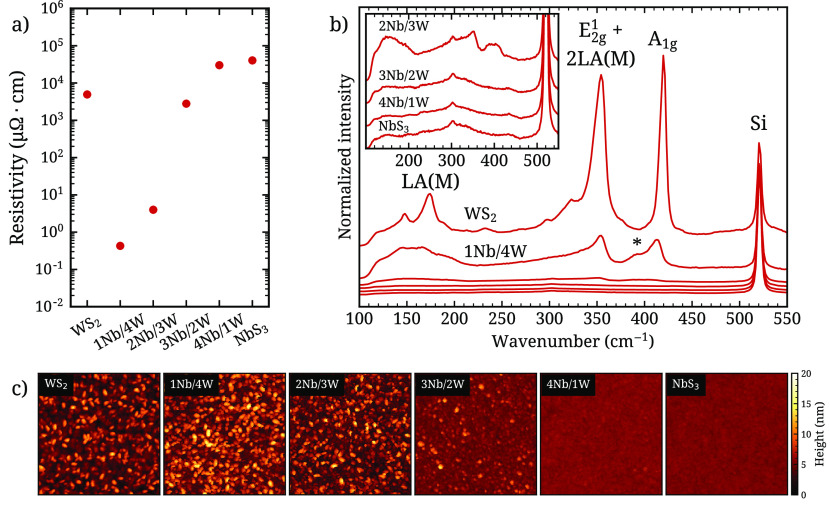
(a) Four-point probe resistivity measurements, (b) Raman spectra and c AFM scans of the Nb_*x*_W_1–*x*_S_*y*_ films (film thickness ∼10 nm, scan dimensions 500 by 500 nm). The arbitrary offsets of the AFM scans in c were chosen such that the mean height of each scan roughly coincides with the middle of the color scale.

Four-point probe measurements (FPP) were conducted to evaluate the electrical resistivity of the Nb_*x*_W_1–*x*_S_*y*_ films grown using the standard supercycle process (see Figure 4a). The pure WS_2_ film has a resistivity of 5 × 10^3^ μΩ cm, which is significantly lower than reported values for CVT-grown bulk crystals (3 × 10^7^ μΩ cm),^16^ sulfurized tungsten metal (1.3 × 10^6^ μΩ cm, grain size <200 nm), and other ALD-grown WS_2_ (1.68 × 10^10^ μΩ cm, grain size <20 nm).^39^ The lower resistivity of our films compared to other ALD-grown films with similar grain sizes could be due to the incorporation of hydrogen in the film since hydrogen radicals are formed in the H_2_S/Ar plasma during processing. As noted before, elastic recoil detection (ERD) measurements confirmed that a relatively high amount of hydrogen H/(H + S + W) ≈ 14 atom % was present in the WS_2_ film. It should be noted that this number is an upper limit for the amount of hydrogen in the film, as it also includes hydrogen that may be present on the sample surface, where a few TFU of hydrogen are typically observed. The ternary film 1Nb/4W has significantly lowered resistivity by 4 orders of magnitude with respect to the pure WS_2_ sample. Further increasing the Nb content of the films is observed to result in an increase in resistivity, with the resistivity of the pure NbS_3_ film exceeding that of the WS_2_ film. This high resistivity of the NbS_3_ film can be understood by its semiconducting nature in combination with its tendency to oxidize in air. In summary, the incorporation of Nb into the WS_2_ film has a strong impact on the film resistivity, as the resistivity of the film can be strongly reduced (4 orders of magnitude) by incorporating Nb.

### Morphology and Crystallinity

To evaluate the crystallinity and morphology of the ternary TMD films, Raman spectroscopy (Figure 4b) and atomic force microscopy (AFM, Figure 4c) scans were carried out on the same 6 samples as represented in Figure 2.

The AFM scans reveal a rough surface for the W-rich films and a transition to a smooth surface for the Nb-rich films. This is in line with the crystalline growth of WS_2_ and the amorphous growth of NbS_3_ at this deposition temperature.^7,24^ The crystalline nature of the W-rich films and the amorphous nature of the Nb-rich films are also supported by the Raman spectra, which show clear WS_2_-like A_1g_ and E_2g_^1^ + 2LA(M) peaks for the W-rich films and no significant features for the Nb-rich films. Notably, this trend in crystallinity aligns with the measured film resistivities (Figure 4a): the rough, crystalline films have a significantly lower resistivity than the smooth, amorphous ones. An exception is the pure WS_2_ film, which, although clearly crystalline as evidenced by the Raman spectra, has a resistivity multiple orders of magnitude larger than the W-rich ternary samples 1Nb/4W and 2Nb/3W. This suggests that both the crystallinity and the incorporation of Nb have a strong impact on the film resistivity.

The Raman spectrum of sample 1Nb/4W has an additional peak at 391 cm^–1^ (labeled * in Figure 4b), which is not observed in the spectrum of the pure WS_2_ sample. To check if this peak is a signature of substitutional Nb incorporation in the WS_2_ lattice, a comparison to Raman spectra from the literature on NbS_2_, NbS_3_, and Nb_*x*_W_1–*x*_S_2_ was carried out. The peak does not appear to originate from 2H-NbS_2_, the Raman spectrum of which is dominated by an A_1g_ mode and an E_2g_^1^ mode, which have been observed experimentally for bulk and thin film samples at Raman shifts of 379 cm^–1^ and 303–309 cm^–1^.^40−42^ This A_1g_ mode at 379 cm^–1^ does not align well with our peak at 391 cm^–1^ and the E_2g_^1^ mode at 303 cm^–1^ is not observed in our measurements (the small peak observed around 300 cm^–1^ for all samples originates from the silicon substrate). 3R-phase NbS_2_ also does not appear to be the origin of the unknown peak: its spectrum is dominated by two A modes and two E modes, which have been observed experimentally at Raman shifts of 386 ± 2 cm^–1^ (A1), 458 ± 3 cm^–1^ (A2), 290 ± 5 cm^–1^ (E1), and 330 ± 3 cm^–1^ (E2) for bulk samples and thin films.^40,43^ Lastly, NbS_3_ does not appear to be the origin of the unknown peak despite its Raman spectrum containing a peak at 387–391 cm^–1^^44,45^ since its spectrum is dominated by stronger peaks around 196 and 340 cm^–1^, both of which are not observed in our spectra. The unknown peak at 391 cm^–1^ is also not observed in the Raman spectra in the Nb_*x*_W_1–*x*_S_2_ literature.^1−3,8,11,14,25^ Its appearance may be related to a high concentration of Nb in the film (35% for sample 1Nb/4W), although the assignment of this peak requires further experiments. Raman spectroscopy was also performed for alloy films with a lower Nb content of 8% prepared with a 4W/1ABC/4W supercycle process. At this low Nb concentration, no new peaks were observed in the Raman spectrum compared to that of pure WS_2_, although the intensity of the resonance Raman peaks was reduced. The spectra are included in the Supporting Information.

**Figure 5:**
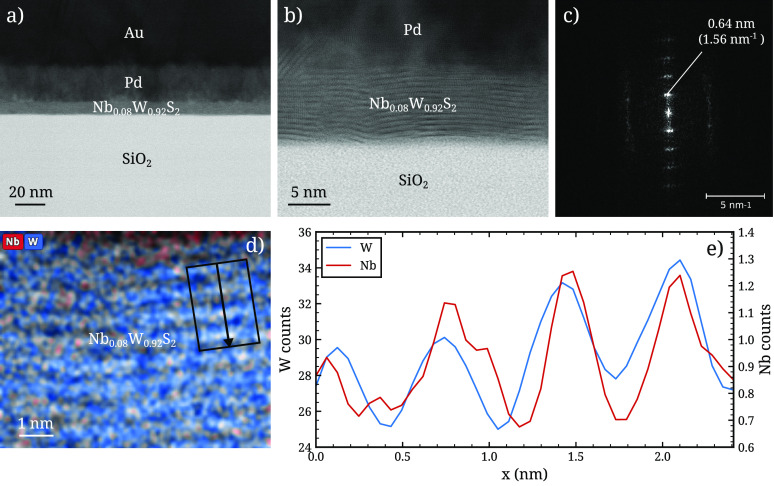
Cross-sectional scanning transmission electron microscopy (STEM) study of a ∼10 nm thick Nb_*x*_W_1–*x*_S_*y*_ with *x* ≈ 0.08 prepared using the 4W/1ABC/4W supercycle process (see Figure 3) on a SiO_2_ substrate. (a and b) show bright-field micrographs of the film cross-section at different magnifications, and (c) shows the 2D Fourier transform of the image shown in (b), from which the interlayer spacing of 0.64 is measured. (d, e) show the spatially resolved composition of the film from energy-dispersive X-ray spectroscopy (EDX). The apparent Nb content at the film surface is suspected to be a measurement artifact due to the low signal-to-noise ratio for the Nb signal. It is unlikely to indicate NbS_*x*_ at the surface since it does not coincide with the S signal.

To further clarify the structure of the Nb_*x*_W_1–*x*_S_*y*_ films and the incorporation of Nb therein, cross-sectional aberration-corrected scanning transmission electron microscopy (STEM) was performed on a sample prepared using the 4W/1ABC/4W process (*x* ≈ 0.08). The bright-field STEM micrographs and energy-dispersive X-ray spectroscopy (EDX) elemental mapping are shown in Figure 5. The film thickness as measured from the micrographs is ∼9 nm, and some surface roughness is observed, which likely explains the slightly larger thickness of ∼10 nm measured by ellipsometry. The layered crystal structure as well as the sulfur–metal–sulfur structure of the layers can be discerned in the high-resolution micrograph in Figure 5c. The interlayer spacing of 0.64 nm is identical to that of pure WS_2_, indicating that the crystal structure of WS_2_ is retained upon incorporation of ∼8% Nb. The EDX elemental mapping of Nb and W in Figure 5d and an extracted line profile in Figure 5e show that the Nb content is high within the WS_2_ layers and low between the layers, suggesting that Nb is incorporated within the layers and not intercalated between the layers. Whether Nb is incorporated substitutionally in the WS_2_ lattice cannot be seen in the present data.

### Electrical Characterization

For electrical characterization, two 10 nm thick films of ALD-grown WS_2_ and Nb_*x*_W_1–*x*_S_2_ (4W/1ABC/4W process) were prepared on p-doped silicon wafers with 90 nm of SiO_2_. The elemental composition of the films was measured by RBS and ERD and is shown in Table 3. Both films have a similar composition except for their Nb content, which is 0% for the WS_2_ film and 8% for the Nb_*x*_W_1–*x*_S_2_ film. In both films, a significant amount of hydrogen is observed at 7.1 TFU (12–14%), which likely originates from the use of H_2_S plasma (which contains hydrogen radicals) in the preparation of the films, as has been reported before.^7^ Similar behavior was also observed in our recent work on the PEALD of MoS_2_.^46,47^ In that work, we have shown that it is possible to mitigate H incorporation during PEALD by using advanced ALD supercycle approaches.^47^ Future studies will aim to minimize unintentional hydrogen content, thus enabling more stable resistivity values.

**Table 3:** Elemental Composition Determined by RBS and ERD of Films Used for Transfer-Length Measurement (TLM) Characterization^a^

sample	Nb/(Nb + W)	S/(Nb + W)	H/(Nb + W + S + H)
WS_2_	0.00	2.24	0.14
Nb_*x*_W_1-x_S_2_	0.08	2.13	0.12

a The spectra are included in the Supporting Information

Back-gated test structures (see Figure S6) were prepared from these two films. The gate voltage sweeps are included in the Supporting Information. In these structures, both films exhibit p-type conductivity, which can be partially attributed to the hydrogen content of the films, as discussed above. However, the measured carrier density of the Nb_0.08_W_0.92_S_2_ film of 3.5 × 10^20^ cm^–3^ is over an order of magnitude higher than that of the WS_2_ film of 1.8 × 10^19^ cm^–3^, indicating that the incorporation of Nb effectively p-dopes the Nb_0.08_W_0.92_S_2_ film. Furthermore, the measured mobility increases significantly from WS_2_ [0.003 cm^2^/(V s)] to Nb_0.08_W_0.92_S_2_ [0.66 cm^2^/(V s)]. It should be noted that the low mobility of WS_2_ can be attributed to the small crystalline grains of the ALD-grown material. The transconductance plots from which these mobility values were extracted are shown in Figure S7. Further performance improvements can likely be obtained by lowering the doping level and reducing the thickness of the film, which are goals for future work.

**Figure 6:**
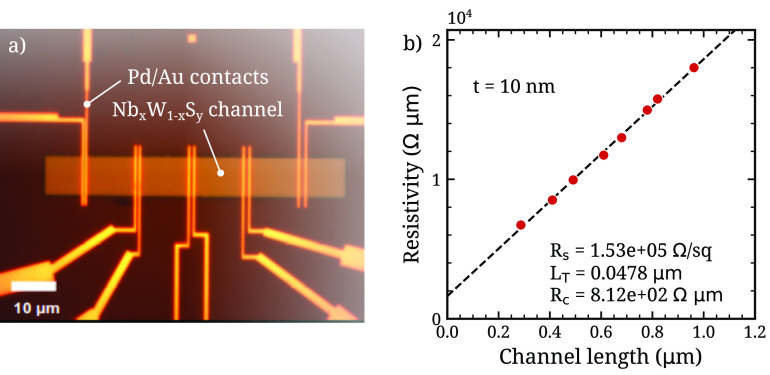
TLM structures (a) of a Nb_0.08_W_0.92_S_*y*_ film of 10 nm thickness deposited on p-doped Si with 90 nm SiO_2_ and (b) results from TLM measurements. Resistance measurements were performed at a gate voltage of 0 V.

Transfer-length measurements (TLM) were performed on the 10 nm Nb_0.08_W_0.92_S_2_ film, as shown in Figure 6. By measuring the resistance as a function of the contact spacing, the resistivity and contact resistance were extracted. For the 10 nm film, a low contact resistance *R*_c_ of 811 ± 104 Ω·μm is observed, which is near the international roadmap for devices and systems (IRDS) target value for the development of 2D TMD transistors (∼300 Ω·μm).^48^ Extracted values of sheet resistance *R*_s_ and transfer length *L*_T_ are also reported in Figure 6. We speculate that the rough surface of the 10 nm film may facilitate electrical contact between the TMD and the metal due to the orientation of TMD edge sites to the contact metal. Additionally, the stability of the Nb_0.08_W_0.92_S_2_ alloy was verified over time through Raman measurements, which are presented in supplementary Figure S10.

In summary, the ALD-grown Nb_*x*_W_1–*x*_S_*y*_ film with composition *x* ≈ 0.08 and thickness 10 nm has a low contact resistivity approaching the IRDS roadmap target value, making it a promising material for contact engineering in 2D TMD transistors.

### Performance as Hydrogen Evolution Catalysts

**Figure 7:**
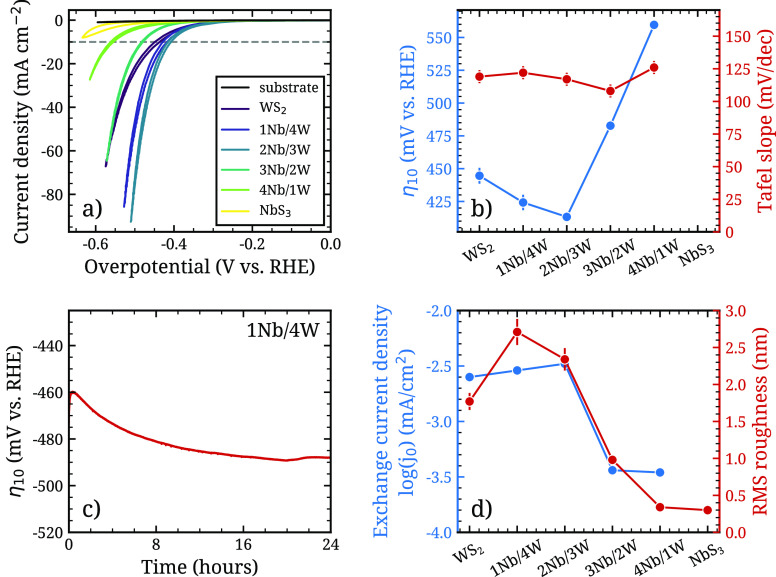
Results of the HER electrocatalysis measurements. (a) Cyclic voltammetry sweeps of the WS_2_, NbS_3_, and alloy samples. The extracted overpotentials at a current density of 10 mA/cm^2^ and Tafel slopes are plotted in (b) Tafel plots are included in the Supporting Information. For the NbS_3_ sample, no Tafel slope or overpotential could be determined, as the Tafel plot did not have a linear region. (c) 24 h stability test of 1Nb/4W deposited on carbon fiber paper. (d) Overlay of the exchange current density obtained from the Tafel plots with the RMS roughness of the samples, as measured by AFM. For the NbS_3_ sample, no exchange current density could be extracted, as the Tafel slope did not have a linear region.

To evaluate the electrocatalytic performance for hydrogen evolution of the Nb_*x*_W_1–*x*_S_*y*_ films, the 6 films discussed in the previous sections were grown on glassy carbon substrates. Their electrocatalytic activity was evaluated in a three-electrode cell using 0.5 M H_2_SO_4_ (pH ≈ 0.3) as the electrolyte. Figure 7a depicts the *iR*-corrected polarization curves from cyclic voltammetry (5th scan). Scans of the bare glassy carbon (GC) substrate are also included to demonstrate that the substrate itself is not significantly catalytically active. Information on scans 1–5 for each sample can be found in the Supporting Information. The overpotential vs reversible hydrogen electrode (RHE) at a current density of 10 mA/cm^2^ (denoted η_10_ from here forward) and the Tafel slope, both common metrics for catalytic activity, were extracted from the curves and used to compare the electrocatalytic performance of the films (see Figure 7b).

The pure WS_2_ film has a η_10_ of 445 mV vs RHE. For the ternary films with 1Nb/4W and 2Nb/3W, this overpotential is lowered to 424 mV vs RHE and 413 mV vs RHE, respectively. Upon further increasing the Nb content, the overpotential increases above that of WS_2_. For the pure NbS_3_, a η_10_ value could not be extracted as the current density did not reach 10 mA/cm^2^ in the voltage range accessed during the measurement. The lower η_10_ values for samples 1Nb/4W and 2Nb/3W indicate that the catalytic activity of WS_2_ is slightly improved by the incorporation of Nb.

The values of the Tafel slope are relatively constant around 120 mV/dec for the W-rich samples WS_2_, 1Nb/4W, and 2Nb/3W. Samples 3Nb/2W and 4Nb/1W deviate slightly from the latter, with values of 108 and 126 mV/dec, respectively. For sample NbS_3_, no regime of linear dependence between the overpotential and current density was observed, so no Tafel slope could be extracted (see Supporting Information for the method). The values of the Tafel slope for the W-rich samples are in line with the theoretical value of 120 mV/dec for an HER catalyst, where the Volmer reaction is the rate-limiting step.^49^

The roughness of samples 1Nb/4W and 2Nb/3W gives them a larger effective surface area compared with the pure WS_2_ sample, which may contribute to their improved catalytic activity. The root-mean-square (RMS) roughness was determined from the AFM images in Figure 4, and the exchange current density determined from the Tafel slopes (see supplementary Figure S9) is shown in Figure 7d. While the roughness of the films on glassy carbon substrates was found to be higher than those on SiO_2_, the trend in roughness with film composition is similar for both substrates, as shown in supplementary Figure S11. The rough surfaces of the crystalline W-rich films are correlated with a larger exchange current density, hence contributing to their catalytic activity. In contrast, the Nb-rich samples both have a smaller RMS roughness and a lower exchange current density (an order of magnitude lower than that of the W-rich films). This suggests that the increased density of active sites due to the larger surface area is a factor in the improved catalytic activity of the crystalline films. Other factors, such as conductivity, likely also contribute to the catalytic activity.

The stability of the 1Nb/4W film under catalytic conditions was investigated by monitoring the overpotential required to maintain a current density of 10 mA/cm^2^ over a time of 24 h (see Figure 7c). After 24 h, a moderate decrease of the overpotential of approximately 30 mV is observed. Furthermore, the change in overpotential seems to saturate within the 24 h of measuring time, such that after this time the overpotential does not appear to change significantly anymore. Hence, after some initial changes within the first hours of measurement, the catalyst appears to stabilize.

In conclusion, our ternary Nb_*x*_W_1–*x*_S_*y*_ HER catalysts 1Nb/4W and 2Nb/3W outperform our pure WS_2_ sample, which we attribute both to increased surface roughness and to activation of the basal plane by incorporation of Nb into the WS_2_ lattice. The same samples also outperform more Nb-rich samples, including pure NbS_3_, which we attribute to the high resistivity and significant oxidation of the Nb-rich samples. The absolute values of the overpotentials are not yet competitive with platinum or high-quality TMD catalysts reported in the literature, and future work will be aimed at further optimizing our catalysts by controlling synthesis parameters.

## Conclusions

In conclusion, we developed a set of plasma-enhanced atomic layer deposition processes for the growth of p-type Nb_*x*_W_1–*x*_S_*y*_ alloys. Using a supercycle recipe, excellent control over the composition x of the alloys is achieved. Through composition tuning, the film resistivity is lowered by 4 orders of magnitude with respect to pure WS_2_ films. The use of advanced supercycles was shown to be effective at reducing the Nb content of the films while maintaining intralayer mixing of Nb and W. A Nb/(Nb + W) fraction of 8% was obtained while maintaining the crystal structure of 2H-WS_2_, as evidenced by Raman spectroscopy, and the 2D layered structure is clearly visible in cross-sectional STEM. The tunable p-type doping level and demonstrated FET characteristics, including low contact resistivity, make ALD-grown Nb_*x*_W_1–*x*_S_*y*_ films of interest for the development of 2D TMD transistors. The Nb_*x*_W_1–*x*_S_*y*_ films also outperform pure ALD-grown WS_2_ and NbS_3_ in the electrocatalysis of the hydrogen evolution reaction, demonstrating the wider applicability of the Nb_*x*_W_1–*x*_S_*y*_ films.

## Experimental Section

Film preparation by plasma-enhanced ALD was performed in an Oxford Instruments FlexAL reactor equipped with ICP plasma in a remote configuration upstream from the substrate table. The table temperature is limited to 350 °C, as decomposition of the Nb precursor was observed at higher temperatures. Due to limited thermal contact between the substrate table and the substrate, the actual substrate temperature is lower than the table temperature and was estimated by in situ ellipsometry to be 290 °C. For pure NbS_3_ and WS_2_ films, deposition processes previously developed by Balasubramanyam et al.^7^ and Basuvalingam^24^ were adopted. The same plasma conditions were used both for WS_2_ and NbS_3_ deposition, i.e., a flow of 40 sccm Ar and 10 sccm H_2_S, a power of 200 W, a pressure of 15 mTorr, and a duration of 30 s. Compared to the original processes, the chamber pressure during precursor dosing was increased from 30 to 200 mTorr to promote the crystallinity of the films.^50^ Nb_*x*_W_1–*x*_S_*y*_ alloys were grown by alternating deposition cycles of WS_2_ and NbS_3_ in a supercycle approach. The W precursor W(N^t^Bu)_2_(NMe_2_)_2_ (BTBMW, 99%, Strem Chemicals) and the Nb precursor Nb(N^t^Bu)(NEt_2_)_3_ (TBTDEN, 98%, Strem Chemicals) are kept in stainless steel canisters heated to 50 °C, and their delivery into the reaction chamber was facilitated with a 50 sccm argon bubbling flow. A saturated precursor dose time of 10 s was used for both precursors. Precursor dosing was followed by a 10 s argon purge of the reactor chamber. As coreactant, a 40:10 sccm Ar/H_2_S plasma of 200 W at a pressure of 15 mTorr was used for 30 s, followed by a 10 s argon purge. For the ABC-type cycles, the adsorption of the Nb precursor was inhibited by preceding its dosing with a saturated (10 s) dose of the W precursor followed by a 10 s argon purge. Silicon wafers with a 450 nm thermal oxide were used as substrates. For the TLM structures, p^+^-doped Si with 90 nm thermal oxide was used. For the HER samples, glassy carbon substrates were used for cyclic voltammetry, and carbon fiber paper was used for stability measurements. Film growth was monitored in situ using spectroscopic ellipsometry using a J.A. Woollam M-2000 ellipsometer in the spectral range of 1.25–4 eV. Film thickness and optical constants were determined by the parametrization of the SE data using a B-spline model.

Atomic force microscopy (AFM) topography scans were made using a Bruker Dimension Icon AFM with a silicon tip PeakForce-air tip on a silicon nitride cantilever, operating in PeakForce Tapping with the ScanAsyst mode.

Scanning transmission electron microscopy (STEM) was performed using a Thermo Fisher Scientific Spectra 300 probe-corrected STEM instrument equipped with a Dual-X EDX system and operated at 300 kV.

Composition analysis was performed with X-ray photoelectron spectroscopy (XPS) using a Thermo Scientific K-alpha spectrometer with an Al K-alpha (1486.6 eV) X-ray source. The binding energy scale was calibrated by shifting the C 1s peak of adventitious carbon to 284.8 eV, in line with the convention in the literature. During deconvolution, the area ratio of doublet peaks is fixed to their physically expected values based on spin–orbit coupling (2:1 for p orbitals, 3:2 for d orbitals, and 4:3 for f orbitals). W 4d spectra are deconvoluted with a W 4d doublet and two singlet peaks attributed to S 2s loss features. W 4f spectra are deconvoluted with two doublets (one attributed to W^4+^) (WS_2_) and the other to W^6+^ (WO_3_) respectively. Nb 3d spectra are deconvoluted using three doublets attributed to Nb^4+^ (NbS_2_), Nb^4+^ (NbS_3_), and Nb^5+^ (Nb_2_O_5_). S 2p spectra are deconvoluted using two doublets attributed to S^2–^ (WS_2_, NbS_2_, and NbS_3_) and S_2_^2–^ (NbS_3_). Deconvoluted spectra and peak parameters are reported in the Supporting Information.

Elastic recoil detection (ERD) and Rutherford backscattering spectrometry (RBS) measurements of composition and areal density of the films were carried out using a 2000 keV He^+^ beam. ERD was performed with a 75° sample tilt and the detector at a recoil angle of 20°. In order to prevent scattered He particles from reaching the ERD detector, a 9 μm thick Mylar stopper foil has been used. With this configuration, it was not possible to separate hydrogen on the surface or interface from hydrogen in the bulk.

Raman scattering spectroscopy was performed with a Renishaw InVia confocal Raman microscope with a 514.5 nm laser, a Leica N Plan EPI 50× objective with a NA of 0.75, and an 1800 lines per mm grating. Each spectrum consists of 10 measurements with an acquisition time of 10 s each and an incident laser power of ∼0.2 mW focused on a ∼1.5 μm spot.

The electrocatalytic activity of the films for the hydrogen evolution reaction (HER) was evaluated in a three-electrode, custom-made glass cell using an Autolab PGSTAT302N potentiostat (Metrohm). The measurements utilized 0.5 M H_2_SO_4_ (pH ≈ 0.3) as the electrolyte, a saturated calomel reference electrode (CH Instruments), and a graphite rod (diameter 6.3 mm, 99%, metal basis, Thermo Scientific) counter electrode. Prior to use in the catalytic measurements, the SCE reference was externally referenced to ferrocenecarboxylic acid in 0.2 M phosphate buffer titrated with NaOH to pH 7 (284 mV vs SCE).^51^ The reference potential determined by this method was then used to convert the measured potentials to the reversible hydrogen electrode (RHE) scale. For this study, thin film samples were deposited on glassy carbon (GC) plates (2.2 × 2.2 × 0.3 cm^2^, SIGRADUR G, HTW Hochtemperatur-Werkstoffe GmbH) and mounted to a rotating disk electrode using a custom-made PEEK sample holder, exposing a 3.14 cm^2^ sample area. Polarization curves were corrected for internal resistance (*iR*-correction) using electrochemical impedance spectroscopy (EIS) measurement and selecting the impedance of the data point with phase closest to 0 for 100% *iR* postmeasurement compensation of all cyclic voltammograms. Afterward, five cyclic voltammetry (CV) scans in the HER region (−0.2 to −0.9 V and back vs SCE) were recorded at a scan rate of 10 mV/s, while the RDE was rotated at 1600 rpm. Tafel slopes and overpotential values (following *iR*-compensation) were extracted from the fifth of these scans. The stability of the 1Nb/4W film on a carbon fiber paper substrate under catalytic conditions, held by a tantalum wire clip (MSE supplies), was investigated by applying a constant current density of −10 mA cm^–2^ geometric surface area and measuring the overpotential required to maintain this current density over a timespan of 24 h.

For electrical characterization, Nb_*x*_W_1–*x*_S_*y*_ mesas were patterned and etched by SF_6_ and O_2_ plasma to form a rectangular active region. Contact stacks of 20 nm Pd and 90 nm Au were deposited by e-beam evaporation, and the contact spacings were measured by scanning electron microscopy (SEM).

## Data Availability

The data that supports the findings of this study are available upon reasonable request from the authors.

## Supporting Information Available

XPS compositional analysis, RBS/ERD spectra, Raman measurements of low Nb content alloys, gate voltage sweeps, cyclic voltammetry sweeps, electrochemical Tafel analysis, long-term air stability of alloy films, and a rms roughness comparison between films deposited on SiO_2_ vs glassy carbon substrates (PDF)
