# Correction to Nb Doping and Alloying of 2D WS_2_ by Atomic Layer Deposition for 2D Transition Metal Dichalcogenide Transistors and HER Electrocatalysts

**DOI:** 10.1021/acsanm.4c02304

**Published:** 2024-05-07

**Authors:** Jeff J. P. M. Schulpen, Cindy H. X. Lam, Rebecca A. Dawley, Ruixue Li, Lun Jin, Tao Ma, Wilhelmus M. M. Kessels, Steven J. Koester, Ageeth A. Bol

The following corrections are reported by the authors. After the corrections, the conclusions drawn in the original are not affected.

Page 7395. In the abstract, the contact resistivity of the 10 nm thick Nb_0.08_W_0.92_S_*y*_ film to Pd/Au contacts was mistakenly reported as (8 ± 1) × 10^2^ Ω cm. The actual value is (8 ± 1) × 10^2^ Ω μm, in line with the TLM results presented in Figure 6. This is unrelated to the following correction.

Page 7400. The resistivity values of the Nb_*x*_W_1–*x*_S_*y*_ films reported in Figure 4a and in the text are mistakenly reported as a factor of 10^4^ lower than their actual values. The corrected Figure 4 is given.

**Figure 4:**
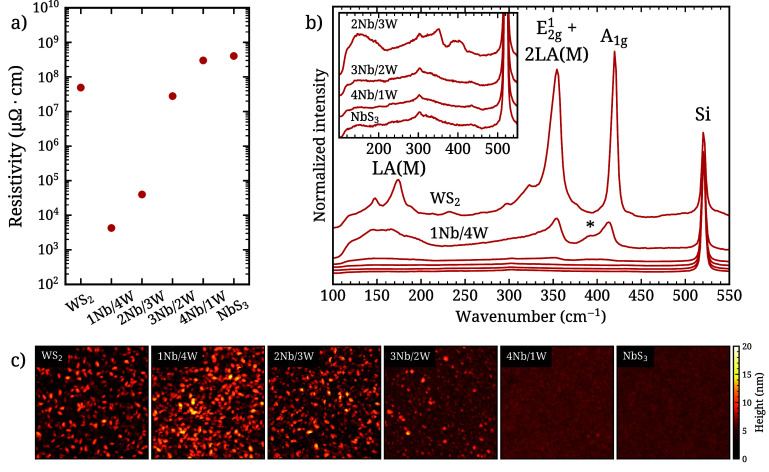
(a) Four-point probe resistivity measurements, (b) Raman spectra, and (c) AFM scans of the Nb_*x*_W_1–*x*_S_*y*_ films (film thickness ∼10 nm, scan dimensions 500 by 500 nm). The arbitrary offsets of the AFM scans in part c were chosen such that the mean height of each scan roughly coincides with the middle of the color scale.

The discussion of the resistivity values changes accordingly as follows:

**Electrical Resistivity**. Four-point probe measurements (FPP) were conducted to evaluate the electrical resistivity of the Nb_*x*_W_1–*x*_S_*y*_ films grown using the standard supercycle process (see Figure 4a). The pure WS_2_ film has a resistivity of 5 × 10^7^ μΩ cm, which is similar to reported values for CVT-grown bulk crystals (3 × 10^7^ μΩ cm),^16^ higher than sulfurized tungsten metal (1.3 × 10^6^ μΩ cm, grain size <200 nm), and significantly lower than other ALD-grown WS_2_ (1.68 × 10^10^ μΩ cm, grain size <20 nm).^39^

The discussion in the remainder of the section regarding the possible role of hydrogen in lowering the film resistivity compared to other ALD WS_2_ reports and the observed trends in resistivity as a function of film composition is unaffected by this correction.

