# Supplementary material for: Nb Doping and Alloying of 2D WS2 by Atomic Layer Deposition for 2D Transition Metal Dichalcogenide Transistors and HER Electrocatalysts
Source: ACS Appl Nano Mater. 2024 Apr 1;7(7):7395–407. doi: 10.1021/acsanm.4c00094 (PMC11019465; doi:10.1021/acsanm.4c00094)
Supplement: Supplementary file 1 — an4c00094_si_001.pdf [file an4c00094_si_001.pdf]

Supplemental Information for: Nb Doping and Alloying of 2D WS_2 by Atomic Layer Deposition for 2D Transition Metal Dichalcogenide Transistors and HER Electrocatalysts

Jeff J.P.M. Schulpen,[1] *Cindy H.X. Lam,*[1] *Rebecca A. Dawley,*[2] *Ruixue Li,*[4] *Lun Jin,*[4] *Tao Ma,*[3] *Wilhelmus M.M. Kessels,*[1] *Steven J. Koester,*[4] *Ageeth A. Bol*[*1,2]

1 Department of Applied Physics, Eindhoven University of Technology, PO Box 513, 5600 MB Eindhoven, The Netherlands

2 Department of Chemistry, University of Michigan, 930 N. University Ave, Ann Arbor, MI, 48109, United States of America

3 Michigan Center for Materials Characterization, University of Michigan, 2800 Plymouth Rd, Ann Arbor, MI, 48109, United States of America

4 Department of Electrical and Computer Engineering, University of Minnesota, 200 Union St Se, Minneapolis, MN, 55455, United States of America

Corresponding author*: Ageeth A. Bol a.a.bol@tue.nl, aabol@umich.edu

XPS peak fitting was performed using Thermo Fisher Scientific Avantage software. For the peak shape, a 30% Lorentzian/Gaussian mix was used. Background subtraction was done using the Avantage “smart background” algorithm which constrains the background to not be higher than the data. For fitting of doublets, the area ratio of the two peaks was constrained to the appropriate value for the orbital quantum number. Figure S1 shows the peak fitting results for the samples in the composition series, and the peak parameters are reported in **Table S1.**

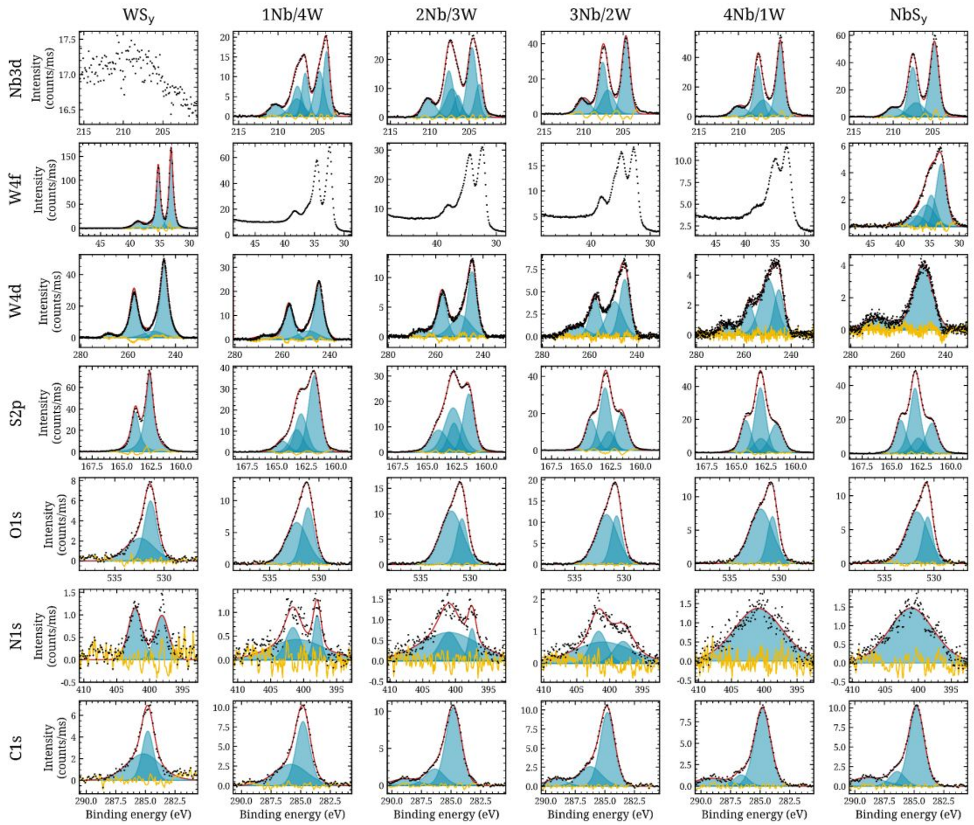

Figure S1: Peak fitting of the XPS elemental scans of the composition series samples. The data is shown as black dots, the fitted peaks in blue, the fitted peak envelope in red and the residual in yellow. The peak parameters are given in **Table S1.**

Table S1: Peak parameters of the XPS peak fits in **Figure S1.**

		WS_y			Nb1W4			Nb2W3			Nb3W2			Nb4W1			NbS_y		
	**Peak**	**BE**	**FWHM**	**Area**	**BE**	**FWHM**	**Area**	**BE**	**FWHM**	**Area**	**BE**	**FWHM**	**Area**	**BE**	**FWHM**	**Area**	**BE**	**FWHM**	**Area**
**Nb3d**	Nb3d5 A	-	-	-	203.86	1.01	18.0	203.74	1.21	15.0	204.65	1.22	58.8	204.7	1.15	67.0	204.68	1.18	67.7
	Nb3d3 A	-	-	-	206.57	1.01	12.0	206.44	1.21	9.96	207.58	1.22	39.1	207.54	1.15	44.6	207.52	1.18	45.1
	Nb3d5 B	-	-	-	204.79	1.4	17.3	204.66	1.39	36.3	206.95	1.87	27.8	206.88	2.18	26.2	206.89	2.43	32.0
	Nb3d3 B	-	-	-	207.57	1.4	11.5	207.59	1.39	24.2	209.95	1.87	18.5	209.88	2.18	17.5	209.89	2.43	21.3
	Nb3d5 C	-	-	-	207.64	2.09	10.0	207.16	1.98	21.1	-	-	-	-	-	-	-	-	-
	Nb3d3 C	-	-	-	210.34	2.09	6.67	210.14	1.98	14.1	-	-	-	-	-	-	-	-	-
**W4f**	W4f7 A	32.86	0.88	97.8	-	-	-	-	-	-	-	-	-	-	-	-	-	-	-
	W4f5 A	35.01	0.88	73.3	-	-	-	-	-	-	-	-	-	-	-	-	-	-	-
	W4f7 B	36.44	1.33	18.8	-	-	-	-	-	-	-	-	-	-	-	-	-	-	-
	W4f5 B	38.51	1.33	14.1	-	-	-	-	-	-	-	-	-	-	-	-	-	-	-
	W4f C	38.7	2.09	7.67	-	-	-	-	-	-	-	-	-	-	-	-	-	-	-
	Nb4p3 A	-	-	-	-	-	-	-	-	-	-	-	-	-	-	-	33.19	2.21	11.2
	Nb4p1 A	-	-	-	-	-	-	-	-	-	-	-	-	-	-	-	35.19	2.21	5.59
	Nb4p3 B	-	-	-	-	-	-	-	-	-	-	-	-	-	-	-	36.37	3.37	3.48
	Nb4p1 B	-	-	-	-	-	-	-	-	-	-	-	-	-	-	-	37.99	3.37	1.74
**W4d**	W4d5 A	244.72	3.72	93.5	244.59	4.39	112	244.58	4.65	55.4	245.16	5.22	36.0	245.19	4.92	16.2	-	-	-
	W4d3 A	257.28	3.72	62.3	257.26	4.39	74.8	257.29	4.65	36.9	257.66	5.22	24.0	257.69	4.92	10.8	-	-	-
	W4d5 B	-	-	-	-	-	-	249.41	9.61	37.4	-	-	-	-	-	-	-	-	-
	W4d3 B	-	-	-	-	-	-	265.47	7.22	10.1	-	-	-	-	-	-	-	-	-
	S2s satt. A	248.19	4.27	23.1	249	9.61	38.0	-	-	-	249.4	8.06	34.0	249.74	8.16	33.1	249.33	10.19	41.8
	S2s satt. B	250.57	11.54	19.3	-	-	-	-	-	-	-	-	-	-	-	-	-	-	-
	S2s satt. C	263.27	14.18	36.7	267.81	8.75	15.0	-	-	-	265.42	9.78	13.2	266.71	8.92	7.56	269.05	12.69	10.3
**S2p**	S2p3 A	-	-	-	161.83	1.18	46.4	161.49	1.1	27.4	161.56	1.12	24.7	161.63	1.3	24.2	161.58	1.25	21.6
	S2p1 A	-	-	-	162.96	1.18	23.2	162.75	1.1	13.7	162.66	1.12	12.4	162.93	1.3	12.1	162.88	1.25	10.8
	S2p3 B	162.48	0.89	48.0	163.32	1.27	14.7	162.82	1.7	32.2	163	1.1	40.3	162.98	1.06	45.1	162.96	1.07	45.0
	S2p1 B	163.69	0.89	21.9	164.55	1.27	7.35	164.06	1.7	16.1	164.2	1.1	20.2	164.23	1.06	22.5	164.19	1.07	22.5
**O1s**	O1s A	531.25	1.45	11.9	531.11	1.48	14.2	530.82	1.28	12.5	530.75	1.28	15.9	530.65	1.23	9.40	530.65	1.21	11.6
	O1s B	532.1	2.66	14.8	532.26	2.59	18.5	531.88	2.66	30.4	531.77	2.56	32.8	531.86	2.77	24.5	531.81	2.64	30.5
**N1s**	N1s A	398.12	2.45	2.63	397.94	1.36	1.38	397.5	1.38	1.18	397.95	3.37	2.51	-	-	-	-	-	-
	N1s B	402.2	2.47	3.09	401.62	2.71	2.01	401.01	3.37	2.56	401.67	2.91	3.16	-	-	-	-	-	-
	Nb3p satt.	-	-	-	401	10.16	4.33	401.29	11.66	7.44	402.41	11.43	7.21	400.93	8.91	12.6	401.04	8.18	12.6
**C1s**	C1s A	284.8	1.39	16.2	284.8	1.4	12.4	284.8	1.56	17.8	284.8	1.45	15.2	284.8	1.61	16.0	284.8	1.53	16.9
	C1s B	285.67	3.37	9.82	285.84	3.3	9.76	286.37	1.83	4.47	286.26	2.21	6.20	286.6	1.42	2.04	286.44	1.52	3.11
	C1s C	-	-	-	-	-	-	288.95	2.63	2.48	289.11	1.93	2.22	289.12	3.37	3.35	288.92	3.37	4.09

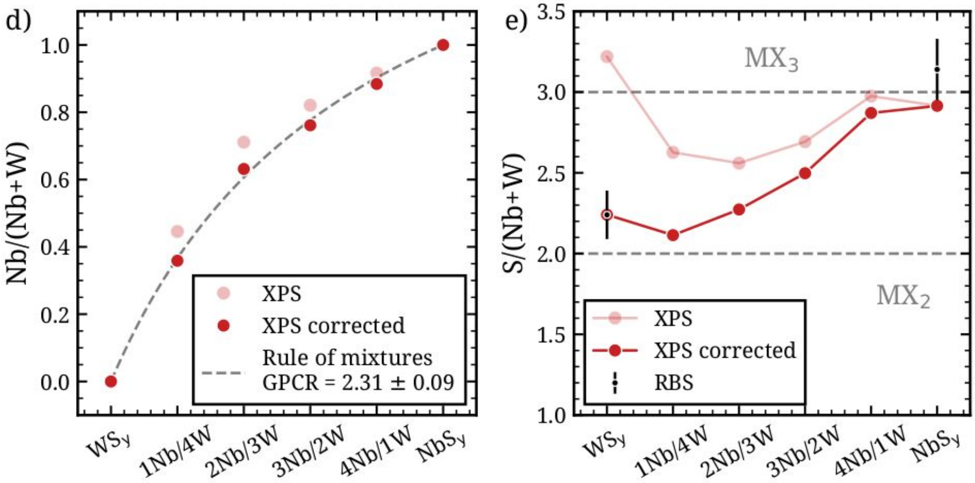

Figure S2: Comparison between XPS results before and after correction of the W quantification as discussed in the main text.

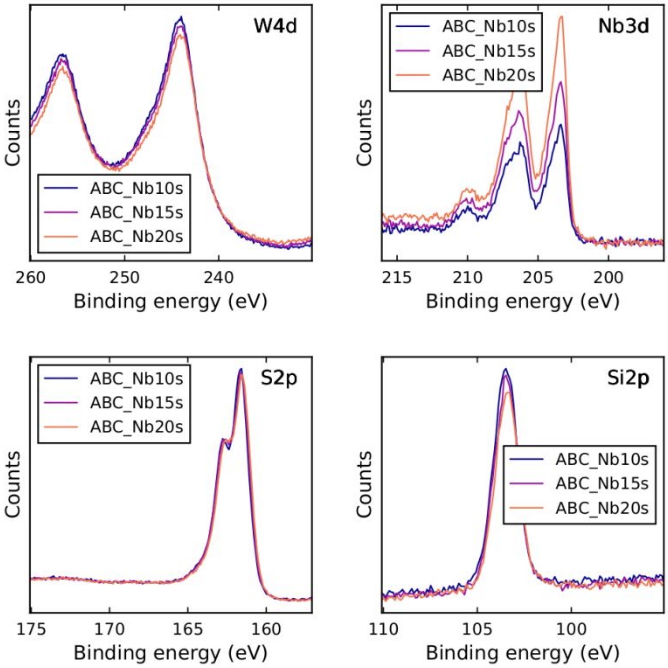

Figure S3: XPS scans for 3 samples prepared using a 4W/1ABC/4W process where the Nb dosetime during the ABC cycle was set to 10, 15 and 20 seconds. This data is represented in numerical form in **Table II**.

RBS and ERD spectra

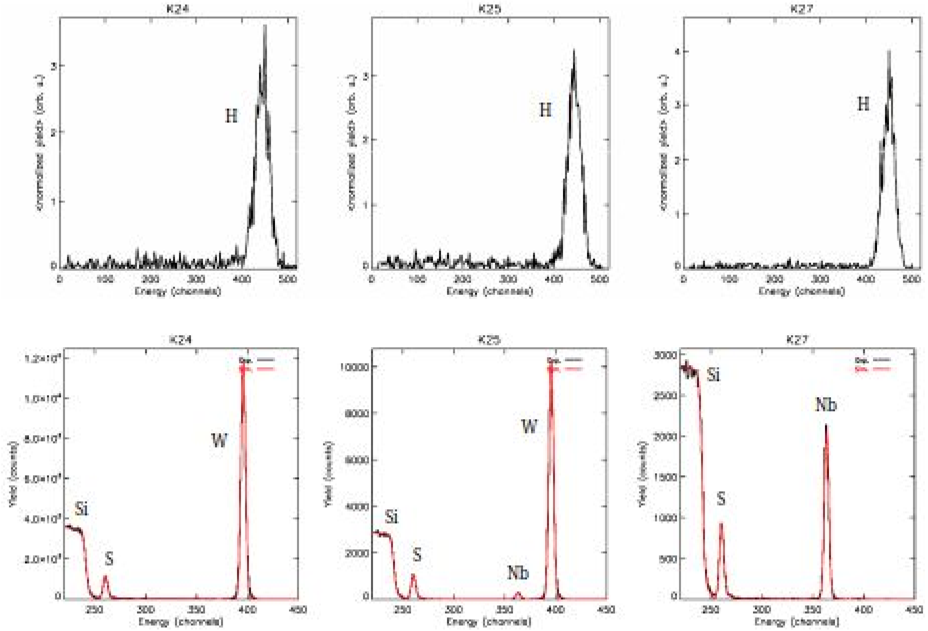

Figure S4: ERD (top) and RBS (bottom) spectra of ALD-grown films WS_2 (K24, left), $Nb_{0.08}W_{0.92}S_2$ (K25, middle) and NbS_3 (K27 right). All three films are 10 nm thick.

The elemental abundance of Nb, W, S and H was derived from the spectra above by fitting, and the results are tabulated below. Units are thin film units (TFU) = 10^{15} atoms per cm^2. Statistical errors are 5% for H, 0.3 TFU for S, 0.1 TFU for Nb, and 1% for W except for sample K27 where it is 20%. On top of the statistical errors, the systematic errors are 7% for ERD (same deviation for H in all samples) and 2% for RBS (same deviation for all elements except H in all samples). The elemental abundance of Nb, W and S was derived from the spectra above by simulation. The hydrogen amounts are obtained by comparison of the peak contents with that of a reference sample of LPCVD-grown silicon nitride.

**Sample**	**Nb (TFU)**	**W (TFU)**	**S (TFU)**	**H (TFU)**
K24	0.0	13.58	30.4	7.10
K25	1.3	14.86	34.5	7.08
K27	9.5	0.016	29.9	6.96

Raman measurements on low Nb content alloys

In the Raman spectrum of $Nb_{0.08}W_{0.92}S_2$, no new peaks are observed compared to the spectrum of the pure WS_2 sample. The new peak observed in the spectrum of the sample 1Nb/4W around 391 cm^{-1} (see main text **Figure 4b**) is not observed either at a low Nb content of 8%. The main difference between the WS_2 and $Nb_{0.08}W_{0.92}S_2$ spectra is a reduced intensity of the resonance Raman peaks, most notably the 2LA(M) peak at 350 cm^{-1}. In contrast, the (nonresonant) A_{1g} peak at 410 cm^{-1} is of similar intensity in both spectra.

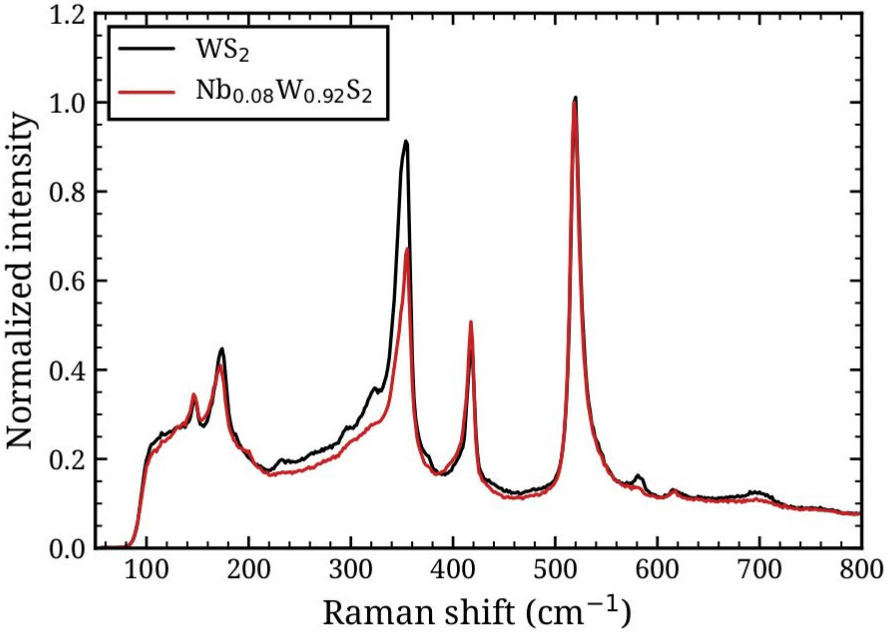

Figure S5: Raman spectra of pure WS_2 and $Nb_{0.08}W_{0.92}S_2$ grown by ALD. For the preparation of the alloy sample, a 4W/1ABC/4W supercycle was used.

Electrical characterization

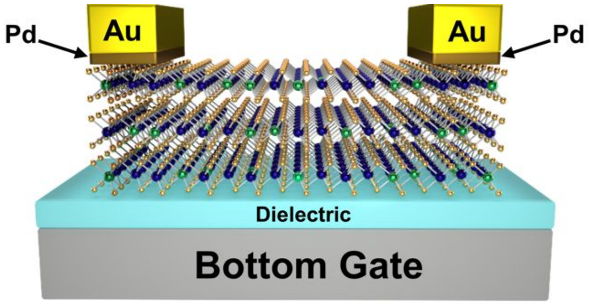

Figure S6: Schematic of prepared back-gated structure used for electrical characterization

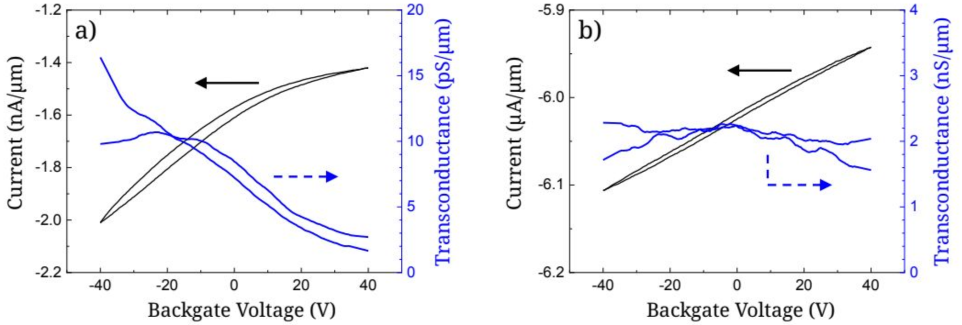

Figure S7: Gate voltage sweeps of backgated test structures based on 10-nm-thick (a) WS_2 and (b) $Nb_{0.08}W_{0.92}S_2$. The contact spacing in each case is 1.2 μm. The backgated oxide was SiO_2 with thickness of 90 nm. The average value of the transconductance between -40 and 0 V was used to extract the carrier mobility in each case, and values of 0.003 cm^2/Vs and 0.66 cm^2/Vs were determined for the WS_2 and $Nb_{0.08}W_{0.92}S_2$ samples, respectively.

Initial cyclic voltammetry sweeps

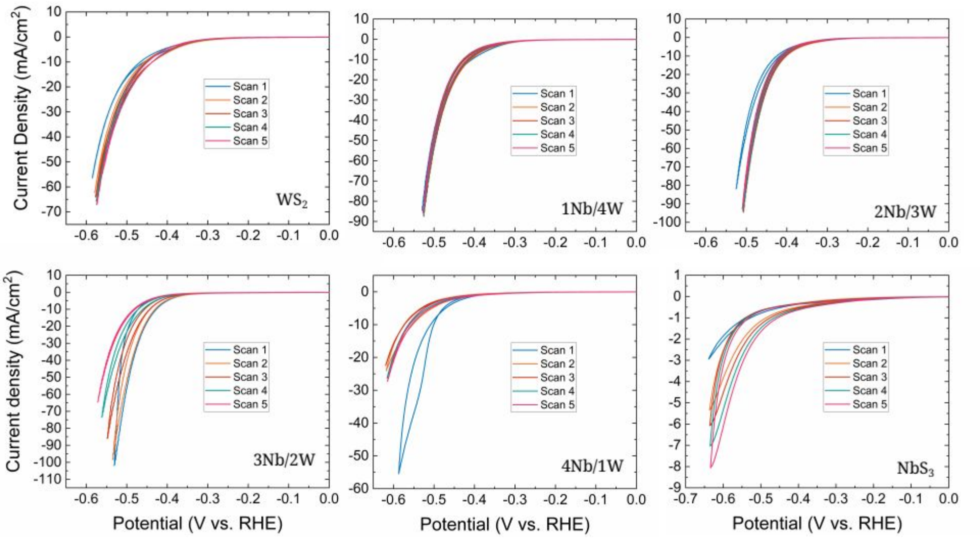

Figure S8: First 5 cyclic voltammetry sweeps of the samples presented in the main text. The fifth sweep was used to determine the Tafel slope and η_{10} overpotential.

Tafel plots and methodology

Tafel slopes were estimated by plotting the numerical derivative of the Tafel plot as a function of the logarithmic current density and identifying the linear part of the Tafel slope by finding the current density where the numerical derivative is (approximately) constant. For the NbS_3 sample, no linear region was found such that a Tafel slope could not be extracted.

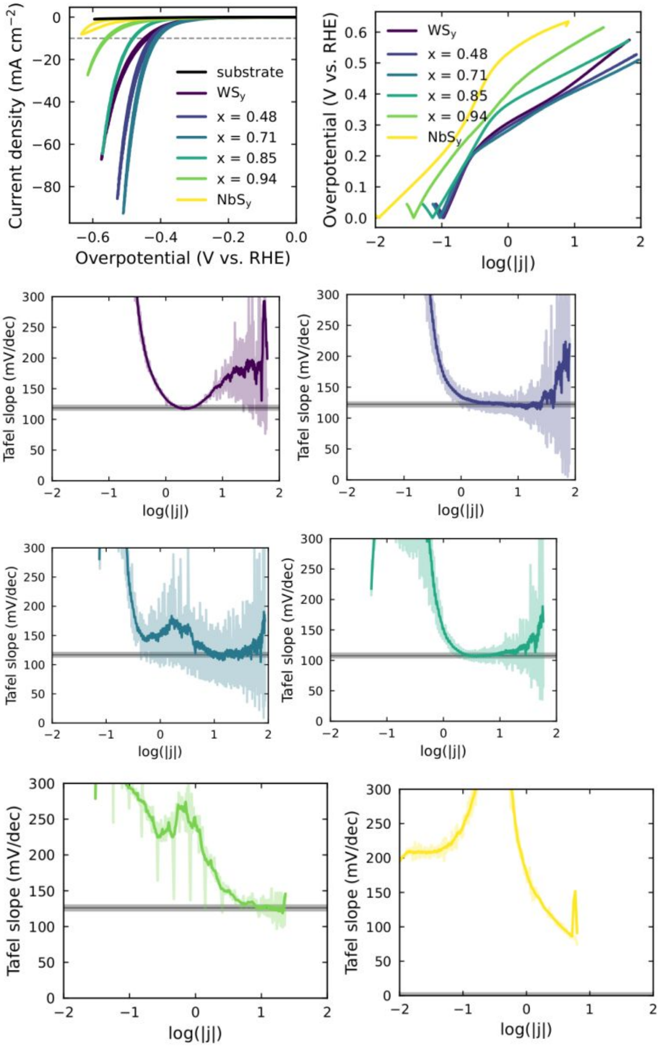

Figure S9: Tafel plots and extraction of Tafel slope values from the linear part of the Tafel plot.

Long-term stability of the $Nb_xW_{1-x}S_y$ films

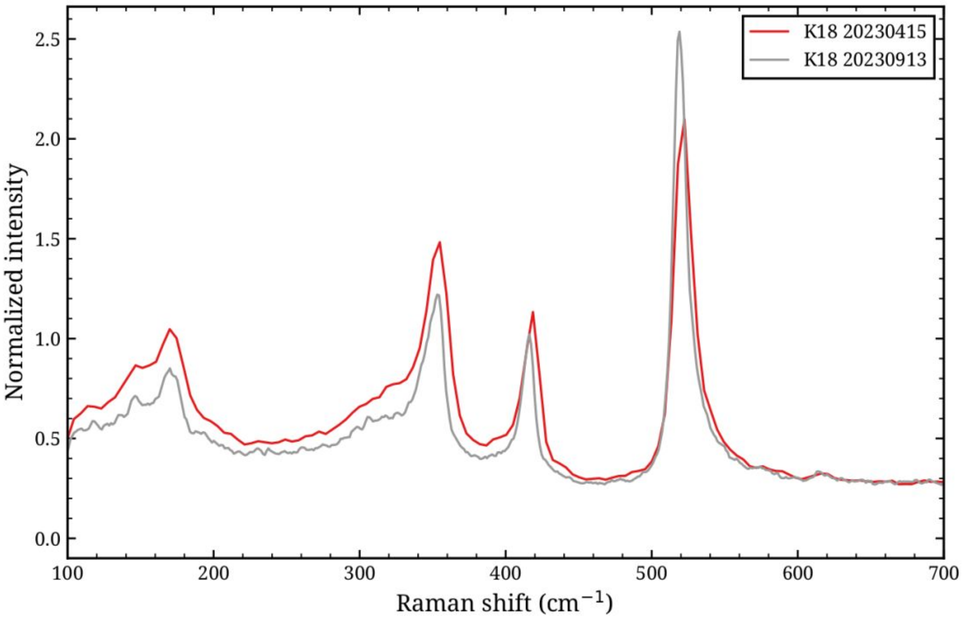

Figure S10: Raman spectra taken on 2023-04-15 and 2023-09-13 of sample K18 ($Nb_{0.08}W_{0.92}S_2$) which was prepared in January 2023. The sample was stored in a vacuum bag in air but was occasionally exposed to air for processing and measurements. While some decrease in intensity of the WS_2-like raman peaks around 350 and 410 cm^{-1} is observed over time, their permanence indicates good overall stability of the material. The spectra are normalized to the mean intensity between 500 and 550 cm^{-1} (the silicon peak). Both spectra were measured on a WiTec Alpha 300R confocal Raman microscope with UHTS300 spectrometer and DV401 CCD detector and a 532 nm laser excitation.

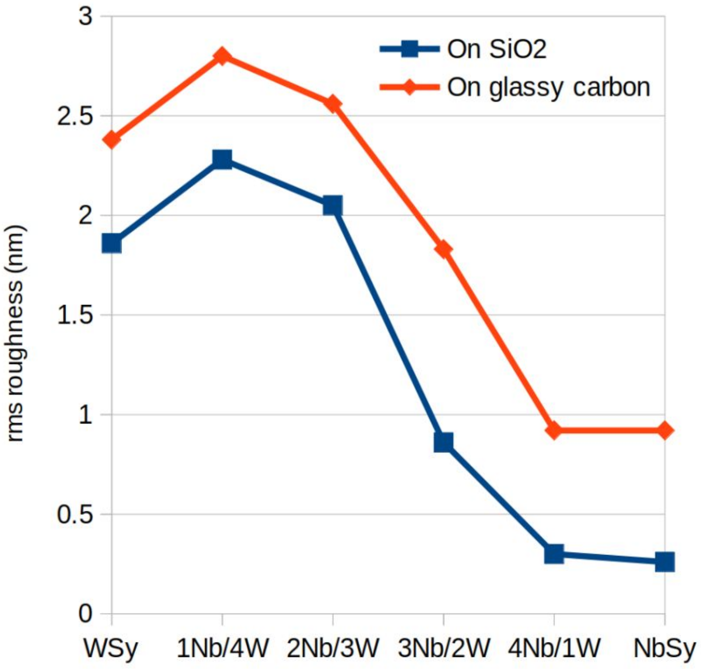

Figure S11: AFM rms roughness values of $Nb_xW_{1-x}S_y$ films on SiO2 and glassy carbon substrates show similar trends in surface roughness with alloy composition on both substrates.
